# Pre-harvest and post-harvest farmer experiences and practices in five maize growing regions in Ghana

**DOI:** 10.3389/fnut.2022.725815

**Published:** 2022-08-19

**Authors:** Bernard Darfour, Kurt A. Rosentrater

**Affiliations:** ^1^Radiation Technology Centre, Biotechnology and Nuclear Agriculture Research Institute, Ghana Atomic Energy Commission, Accra, Ghana; ^2^Agricultural and Biosystems Engineering Department, Iowa State University of Science and Technology, Ames, IA, United States

**Keywords:** pre-harvest losses, post-harvest losses, maize grain storage, Ghana, storage

## Abstract

Maize is a major staple crop mainly produced by smallholder farmers in developing nations. Grain losses happen in Sub-Saharan Africa, and therefore the objective of this study was to assess the different kinds of pre-harvest and post-harvest losses that maize farmers in Ghana encounter. The storage practices, and farmers' awareness and knowledge of mycotoxin contamination in maize were also assessed. The study area had five regions, and three districts per region. The study sites were selected purposefully because of the prior knowledge of farmers on maize production. A semi-structured questionnaire was used to collect the data, and a purposive sampling technique was used to select 75 maize farmers for the interview. The male maize farmers were many compared to females. Over 70% of farmers were at least 40 years. Over 50% of farmers had basic education except those in the northern region. Grain yields were generally low, and at least 60% of farmers experienced post-harvest loss. The period of grain storage and the storage techniques were the prerogatives of the farmers but largely dependent on farmers' financial status. Farmers basically used synthetic chemicals, and a few of the farmers decided to use plant materials during grain treatment.

## Introduction

Maize is a major crop in the agricultural sector of Ghana, and also as a food security crop. Maize crop is well adapted, and grown in all the agro-ecological zones in Ghana (1, 2). However, they are dominantly grown in the middle to southern zones (3). Maize production is about 31% of the food crops under cultivation, and 70% of the total cereal production in Ghana (2). Maize is a staple crop which is highly used at the household level compared to industrial scale. Maize consumption accounts for over 25% of calories consumed of which about 75% comes from local production (2). The high demand for domestic consumption has resulted in increased maize production. Therefore, the production volume of maize had increased from 2 million metric ton to 3.1 million metric ton between 2017 and 2020 (4).

Maize grains undergo various cleaning processes before storage. To extend the shelf-life of stored grain, grain are protected from pests and other unfavorable environmental conditions (5). For decades, many traditional methods have been used to keep grains in safe storage. However, other modern and advanced post-harvest techniques contemporary exist. In 2012, post-harvest loss (PHL) in maize in Ghana was as high as about 70% (6). There are primary, secondary and tertiary factors which can result in PHL in maize (7). The primary factors may include genetic, pest and diseases, physiological, environmental, storage infrastructure, and processing. The secondary factors may include road network accessibility, transportation, market information, infrastructure, knowledge, and consumer behavior. Tertiary factors may include policy of government, investments in agriculture, advocacy groups, and participation by private sector among others. The primary, secondary and tertiary factors interact to determine the extent of PHL.

Mycotoxin contamination in maize grain has human and animal health issues, and maize quality and quantity loss issues which are major challenges in Ghana. To improve food and nutrition security, molds contamination and PHL in grains should be reduced. This is because the loss in grain quality and quantity increases food prices and reduces access and availability of nutritious food. Farmer's purchasing power reduces and poverty level then rises (8). PHL in SSA is still high although steadily decreasing, therefore the aim of this study was to assess the different kinds of pre-harvest and post-harvest losses that maize farmers in Ghana encounter. The storage practices, and farmers' awareness and knowledge of mycotoxin contamination in maize were also assessed.

## Methodology

### Study area and administering of interviews in Ghana

**Figure 1:**
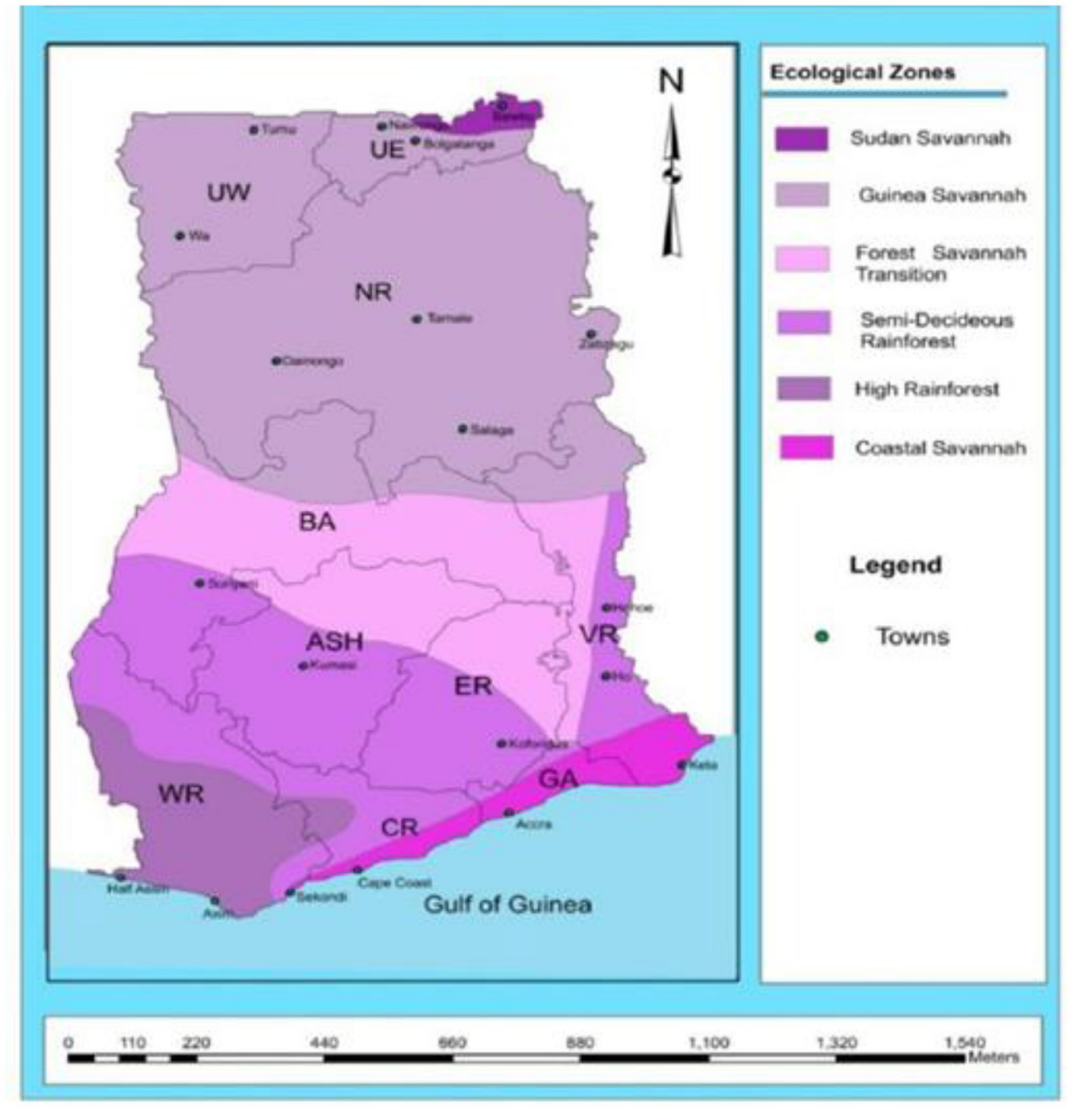
Ecological zones of Ghana [source: Issaka et al. (12)].

A pre-survey was tested in Akuapim South District in mid-May, 2016. See Supplemented material for actual survey questions asked of the farmers. The actual study area had five regions, and each region had three districts (Table 1). The study sites were purposely selected because those areas have larger population of maize farmers who cultivate maize in relatively larger acreage (3, 5, 9–11). The agro-ecological zones within which this study happened are found in Figure 1. The differences between the agro-ecological zones are based on the amount of rainfall, differences in soils, and general crops found in that zone. A semi-structured questionnaire was used to collect the data. A purposive sampling technique was used where 75 maize farmers were judgmentally selected and interviewed between May and August 2016 through the efforts of their Agricultural Extension Agents (AEAs). Questions were asked on the major causes of pre-harvest and post-harvest losses in maize, methods and length of maize storage, grain handling practices, knowledge of mycotoxin contamination, etc. Questions were asked in participants' local language.

**Table 1:** Regions and districts where the interviews were administered to maize farmers.

**Regions**	**District/Municipality**		
	**1**	**2**	**3**
Central Region (CR)	Agona East	Asikuma-Odoben-Brakwa	Gomoa East
Eastern Region (ER)	Akuapim North	East Akim	Kwahu East
Ashanti Region (AR)	Asante Akim South	Ejura-Sekyedumase	Ejisu-Juaben
Brong Ahafo Region (BA)	Kintampo North	Nkoranza South	Techiman
Northern Region (NR)	Tolon	Kumbungu	Tamale

### Data presentation

The data were coded and calculated in percentages. The data were presented in both tabular and graphical form. The data trends were described using descriptive statistics.

## Results and discussion

### Demographic information on farmers

Based on this study, the male farmers in all the five regions were more than their female counterparts (Table 2). This result contradicts the perception that female farmers in Ghana outnumber their male counterparts. Jolly et al. (13) similarly indicated that male farmers in Ghana outnumber females. Contrary, research in Tanzania and Nigeria have reported a high percentage of female farmers compared with male farmers (14–16). Women in agriculture (%) found in SSA and other developing countries ranges between 43 and 50% (17). Based on the regions surveyed, more than 60% of farmers were at least 40 years old. The youth have shown lack of interest in farming, and therefore, most farmers are above 40 years. If this trend continues across all the regions, it can affect the agricultural workforce in Ghana in the near future. This can lead to limited food supply and massive food insecurity and poverty.

**Table 2:** Demographic data of the respondents.

**Parameter**		**Percentages**				
		**Central region**	**Eastern region**	**Ashanti region**	**Brong Ahafo region**	**Northern region**
Gender	Males	93.0	73.0	73.0	80.0	73.0
	Females	7.0	27.0	27.0	20.0	27.0
Age group (Years)	18–25	0.0	6.7	0.0	0.0	0.0
	25–40	33.3	6.7	0.0	26.7	26.7
	Over 40	66.7	86.7	100.0	73.3	73.3
Educational level	None	0.0	0.0	13.3	46.7	86.7
	Primary	6.7	26.7	26.7	20.0	13.3
	Middle	6.7	20.0	6.7	6.7	0.0
	Secondary	80.0	46.7	13.3	26.7	0.0
	College	6.7	6.7	40.0	0.0	0.0

At least 50% of farmers have had primary education, except farmers in NR. The number of farmers without formal education was highest in BA (47%) and NR (87%). The low level of education in the NR can be attributed to lack of schools within the farming communities some years ago. Because of illiteracy especially in NR, farmers depended mostly on their AEAs and colleagues for information. The farmers with higher levels of education were regular formal sector workers who farm as secondary job. Similarly, Suleiman et al. (16) reported that in Tanzania most maize farmers completed at least primary education.

### Cultivated farm size, and grain handling before, during, and after harvest

Unlike the wives of these male farmers who cultivated an acre of land, most single-mothers cultivated 2–3 acres. This was to help them meet their financial obligations through the sale of some of the farm produce. About 73% of farmers cultivated 2–5 acres of land in BA, CR, and ER while it was 67 and 53%, respectively, in AR and NR (Table 3). A fewer percentage of farmers (20%) cultivated between 6 and 10 acres of land in BA and NR. The farmers who participated in the study were typically smallholder farmers since their farm sizes were in smaller acreages. According to FAO (17), smallholder farmers cultivate at most 25 acres (10 ha) of land.

**Table 3:** Farm sizes cultivated by maize farmers, and the different methods of harvesting maize grain.

**Parameter**		**Percentages**				
		**Central region**	**Eastern region**	**Ashanti region**	**Brong Ahafo region**	**Northern region**
Area cultivated in acres	<1	6.7	0.0	0.0	0.0	0.0
	1	0.0	13.3	13.3	6.7	26.7
	2–5	73.3	73.3	66.7	73.3	53.3
	6–10	13.3	13.3	13.3	20.0	20.0
	>10	6.7	0.0	6.7	0.0	0.0
Means of harvesting	Hand	100.0	100.0	100.0	100.0	100.0
	Mechanized	0.0	0.0	0.0	0.0	0.0

Farmers experiencing pre-harvest loss was at least 93% (Table 4) while pre-harvest loss in maize grain was <1%, but it was 2% in NR. There were variations in the causative agents and extent of damage associated with pre-harvest loss amongst the regions. Generally, birds, weather, insects, plant lodging, and rodents were the causative agents. Plant lodging was rampant since it was caused by the blowing wind (uncontrollable), rodents and termites. The low level of pre-harvest loss possess less concern regarding food insecurity.

**Table 4:** Pre-harvest loss and associated casual agents experienced by the maize farmers.

**Parameter**		**Percentages**				
		**Central region**	**Eastern region**	**Ashanti region**	**Brong Ahafo region**	**Northern region**
Pre-harvest loss	Yes	100.0	100.0	100.0	93.3	93.3
	No	0.0	0.0	0.0	6.7	6.7
Agents of pre-harvest loss	Birds	19.4	25.6	34.3	32.1	26.7
	Weather	19.4	23.3	25.7	28.6	6.7
	Insects	45.2	30.2	34.3	25.0	36.7
	Rodents	16.0	20.9	5.7	14.3	30.0
Level of loss	<1	60.0	46.7	66.7	80.0	0.0
	2	6.7	13.3	20.0	20.0	80.0
	>3	33.3	40.0	13.3	0.0	20.0

All farmers typically harvested maize using handheld machete (Table 3) due to lack of combine harvesters. The manual harvesting leads to longer days used in harvesting, thus up to 2 weeks (Table 5). The longer days used in harvesting might result in higher field loss in grain quality and quantity caused by molds and weevils. Farmers that cultivated larger acres of land had to hire casual laborers during the harvesting period. Grain yields were generally low across all the regions. Thus, CR (20%) and BA (27%) of farmers had between 500 and 3,000 kg of grain. ER (86%) had 1,500 kg, AR (60%) recorded 500–3,000 kg, and AR (33%) above 3,000 kg. NR (93%) recorded between 500 and 3,000 kg. Such low yield are attributable to non-mechanized farm operations, rain-fed farming, and inability to purchase farm supplies and inputs (fertilizers, herbicides, pesticides, insecticides, machetes, etc.). Farmers complained about unavailable financial loans and limited government subsidies on agriculture inputs and supplies. Mechanizing farming and government increasing subsidies on agriculture inputs can help increase maize grain production in Ghana. Similarly, Abass et al. (18) observed a lower yield among maize farmers in Tanzania, and they attributed it to manual farming operations. In order to avoid the hustle associated with post-harvest handling of grain, most farmers in ER sold the maize before physiological maturity (green stage) instead of allowing the grain to dry. This practice, however, is a recipe for food insecurity since scarcity and price of grain can increase.

**Table 5:** Farmers' experience in maize grain harvesting, threshing, and storage.

**Parameter**		**Percentages**				
		**Central region**	**Eastern region**	**Ashanti region**	**Brong Ahafo region**	**Northern region**
Number of days used in harvesting	<4 days	66.7	60.0	33.3	33.3	60.0
	1 week	33.3	20.0	20.0	66.7	40.0
	2 weeks	0.0	20.0	26.7	0.0	0.0
	>2 weeks	0.0	0.0	20.0	0.0	0.0
Number of bags per harvest (100 Kg)	<5	6.7	33.3	6.7	13.3	0.0
	5–15	46.7	53.4	33.3	40.0	46.7
	15–30	20.0	13.3	26.7	26.7	46.6
	>30	26.6	0.0	33.3	20.0	6.7
Methods used in grain threshing	Hand	13.3	26.7	0.0	13.3	20.0
	Mechanized	0.0	0.0	40.0	73.4	33.3
	Improvised device	0.0	6.7	13.3	0.0	0.0
	Beating	13.3	33.3	6.7	13.3	26.7
	Beating and hand	73.4	33.3	40.0	0.0	20.0
Number of months of grain storage	<1 month	0.0	0.0	0.0	6.7	0.0
(domestic use)	3 months	13.3	20.0	33.3	33.3	13.3
	6 months	20.0	13.3	6.7	40.0	33.3
	12 months	66.7	66.7	60.0	20.0	53.4

**Figure 2:**
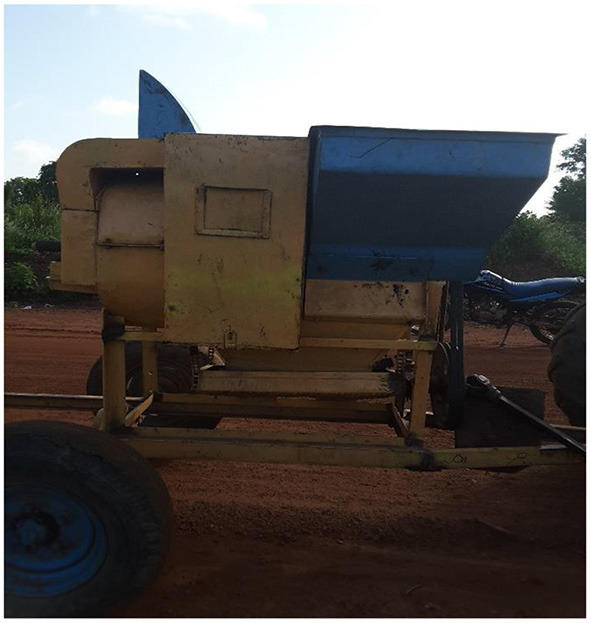
Locally manufactured thresher used in the Northern Region of Ghana.

**Figure 3:**
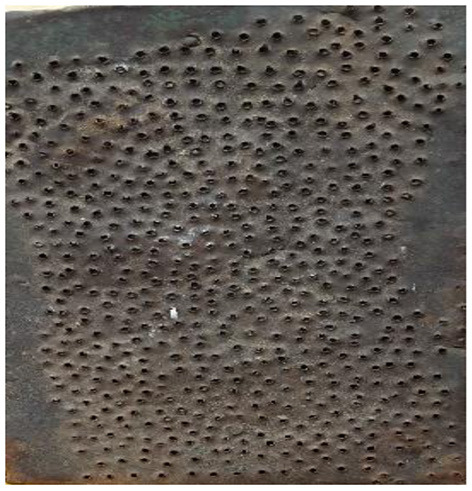
Improvised tool for shelling maize grain.

Farmers threshed grain using their hand, beating or the combination of hand and beating methods. As in Table 5, farmers had preferred apparatus for grain threshing. Mechanical threshing was available although limited in BA, AR and NR, where 73, 40, and 33% of farmers, respectively used. The challenge associated with the use of the locally manufactured thresher (Figure 2) was the lower efficiency in threshing which results in grain loss and damage. Some farmers used improvised tools (Figure 3) during shelling. The use of threshers with higher efficiency rather than improvised tools can greatly reduce grain loss and damage. In support of this study, Abass et al. (18) reported that farmers in Tanzania shell maize grain manually.

### Post-harvest grain drying, sorting, and storage

**Figure 4:**
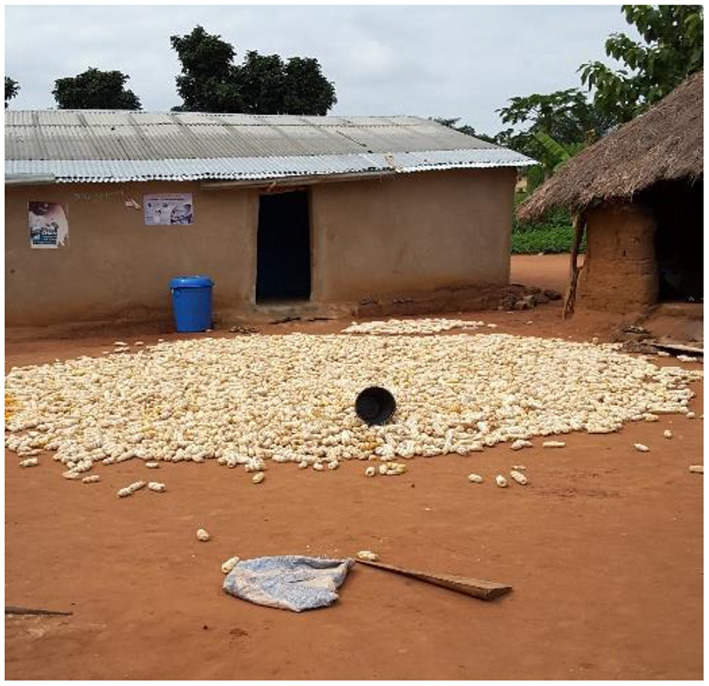
Drying of shelled grain on a bare floor in Brong-Ahafo region.

The months for storing grain was the prerogative of the farmers which varied from weeks to 12 months (Table 5). Farmers sold the grain as and when they are in need of income. Not <93% of farmers in all the regions used sun drying (Table 6). Farmers use sun drying because the energy from the sun is predominant in Ghana, and a cost-free source of energy. Interestingly, farmers sun-dry the grain lot mostly on a bare floors or cemented pads or mats or tarpaulins or large polyethylene which exposes the grain to birds, foreign contaminants, livestock, and unexpected rainfall (Figure 4). All the above can reduce grain quantity and quality, and hence, increase food insecurity and poverty among farmers. Moreover, in the AR, 7% of farmers used solar drying (Table 6). The solar dryer was manufacture from solar tent which has the ability to trap solar radiation to help dry the grain. The solar drying tent essentially maintains grain quality and quantity.

**Table 6:** Post-harvest techniques practiced by maize farmers.

**Parameter**		**Percentages**				
		**Central region**	**Eastern region**	**Ashanti region**	**Brong Ahafo region**	**Northern region**
Insects infection	Weevil	100.0	100.0	100.0	100.0	100.0
Types of drying used	No drying	26.7	6.7	0.0	13.3	0.0
	Sun drying	73.3	93.3	93.7	86.7	100.0
	Solar drying	0.0	0.0	6.3	0.0	0.0
	Mechanical drying	0.0	0.0	0.0	0.0	0.0
Sorting of foreign materials	Yes	100.0	80.0	93.3	80.0	100.0
	No	0.0	20.0	6.7	20.0	0.0
Sorting of insects	Yes	100.0	80.0	93.3	80.0	100.0
	No	0.0	20.0	6.7	20.0	0.0
Sorting of broken seeds	Yes	86.7	66.7	93.3	80.0	100.0
	No	13.3	33.3	6.7	20.0	0.0
Methods used in sorting	Hand	20.0	6.7	6.7	13.3	6.7
	Winnowing	0.0	6.7	0.0	0.0	6.7
	Mechanical	0.0	0.0	0.0	0.0	0.0
	Hand and Winnowing	80.0	66.6	86.6	66.7	86.6
	No sorting	0.0	20.0	6.7	20.0	0.0
Means of storage	Granary	33.3	56.3	20.0	0.0	0.0
	Silo	0.0	0.0	0.0	0.0	0.0
	Polypropylene bag	13.3	37.5	60.0	86.7	100.0
	Granary and bags	53.4	6.2	20.0	13.3	0.0

Over 67% of farmers practiced grain sorting where foreign materials, mold instead or insect infested kernel, broken kernels, broken cob, etc. were removed. Those farmers practically used handpicking or winnowing method. The inefficient way of grain sorting resulted in farmers recording higher rates of grain deterioration. Grain sorting reduces grain spoilage (19), and also maintains grain quantity, quality, and grade.

**Figure 5:**
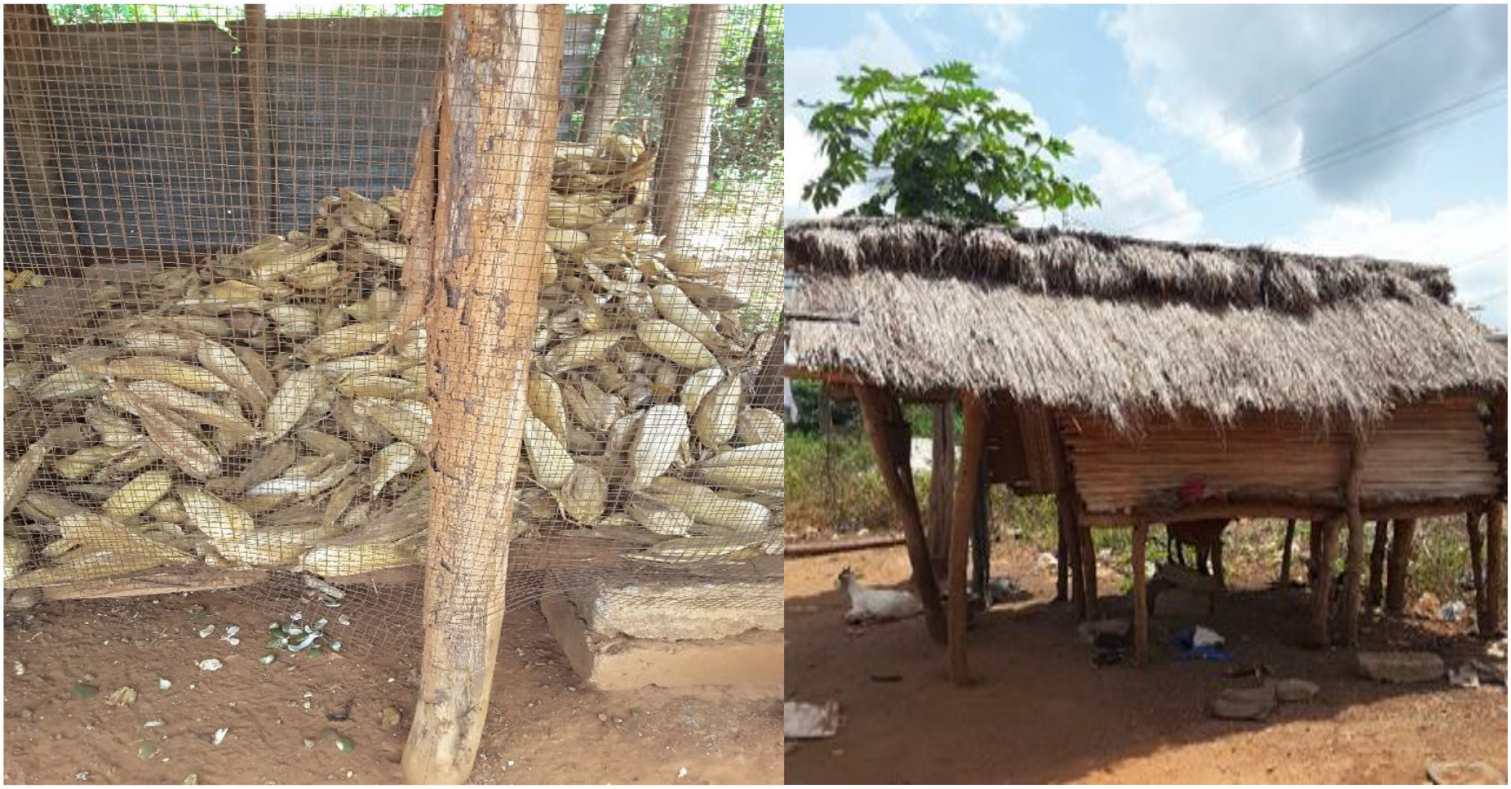
Traditional granaries used to store maize by some farmers.

Some farmers still use traditional granary and/or and polypropylene bags (Figures 5A,B). At least 60% of farmers in AR, BA, and NR used polypropylene bags to store grain. The traditional granary which is susceptible to insects and rodents attack was extensively used in ER and CR. A few farmers used hermetic storage techniques which keep grain quite safe in storage. But farmers were not using the hermetic techniques, and therefore PHL in grain was high. Most farmers (87%) tend to sell their entire grain just after harvest, and keep a little for domestic consumption. Farmers in CR, ER, AR, and BA stored grain for a maximum of 6 months because of the two-seasonal cultivation periods experienced each year.

### Post-harvest grain treatment

**Figure 6:**
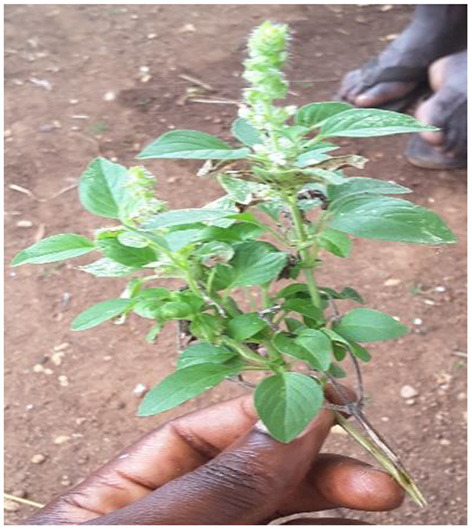
Basil herbal plant used by some farmers to treat maize grain.

About 87% of farmers in CR used synthetic insecticides in grain treatment (Table 7). In ER and AR, about 50% of farmers either used or did not use synthetic insecticides. While in BA 20% of farmers used synthetic insecticides, and 40% in NR used fumigants. Some farmers preferred using plant materials to treat grain (NR, 87%; ER, 27%; and AR, 7%). A few farmers used synthetic insecticides in BA because most farmers sold out grain days or weeks just after harvest. Hence chemical treatment was unnecessary. Most farmers in NR used plant materials to treat grain, hence low synthetic insecticides (fumigants) application. Some farmers in NR used a combination of fumigants and plant materials during grain storage. The common insecticides used by farmers were actellic super, phosphine (fumigant), etc. The predominant use of synthetic insecticides can partly be linked to their market availability and relatively lower price compared with other botanicals. The common plant materials used by farmers that participated in the interview included neem, red hot pepper and basil plants (Figure 6). The farmers claimed that the plant materials used were effective against maize weevils, although they did not extract the essential oils. Many studies have reported the potency of insecticidal essential oils extracted from different plant materials against insects (20–23).

**Table 7:** Different treatment types used by farmers for storage of shelled maize grain.

**Parameter**		**Percentages**				
		**Central region**	**Eastern region**	**Ashanti region**	**Brong Ahafo region**	**Northern region**
Usage of chemical treatment	Yes	86.7	46.7	53.3	20.0	40.0
	No	13.3	53.3	46.7	80.0	60.0
Usage of herbal treatment	Yes	0.0	26.7	6.7	0.0	86.7
	No	100.0	73.3	93.3	100.0	13.3
Chemical used in treatment	Insecticides	100.0	100.0	100.0	100.0	0.0
	Fungicides	0.0	0.0	0.0	0.0	0.0
	Fumigants	0.0	0.0	0.0	0.0	100.0

### Mold infestation, knowledge of farmers, and AEAs' activities

In Table 8, the percentage of farmers that experienced post-harvest mold attacks ranged from 13 to 40%, and BA recorded the highest. The higher molds infestation in BA can be associated with higher rainfall pattern in BA. This is because molds thrive on wet or damp grain (24), especially when the process of harvesting and/or drying is delayed. As indicated by Gbangou et al. (25), southern and transition zones have the highest average seasonal rainfall. At least 90% of all the participated farmers had knowledge of the effects of using moldy grain. Farmers knowledge of the effects of mycotoxin on humans and animals health were 93, 60, 60, 27, and 20%, respectively, in NR, AR, BA, ER, and CR. Despite the low formal education level of farmers in NR, their knowledge of mycotoxin was extensive. This was basically because of the regular education provided by the AEAs. Surprisingly, most educated farmers lacked knowledge of mycotoxin and its effects. In Malaysia, Leong et al. (26) similarly found no significant association linking knowledge of aflatoxin to education level of farmers. Notwithstanding, contrary findings were reported by Jolly et al. (27) and Suleiman et al. (16). The efforts of the AEAs were greatly acknowledged by not <73% of farmers across all regions. An example of such efforts was observed in NR regarding farmers' knowledge of mycotoxin.

**Table 8:** Farmers' knowledge of mycotoxins, and acknowledgment of AEAs' activities.

**Parameter**		**Percentages**				
		**Central region**	**Eastern region**	**Ashanti region**	**Brong Ahafo region**	**Northern region**
Observation of fungi infestation	Yes	26.7	20.0	13.3	40.0	20.0
	No	73.3	80.0	86.7	60.0	80.0
Knowledge of effects of moldy grains	Yes	93.3	93.3	93.3	93.3	93.3
	No	6.7	6.7	6.7	6.7	6.7
Previously heard of the word mycotoxins	Yes	6.7	26.7	60.0	6.7	93.3
	No	93.3	73.3	40.0	93.3	6.7
Aware of mycotoxins contamination in maize	Yes	20.0	26.67	60.0	60.0	93.3
	No	80.0	73.33	40.0	40.0	6.7
Aware of effects of mycotoxins on human and animals	Yes	20.0	20.0	60.0	46.7	93.3
	No	80.0	80.0	40.0	53.3	6.7
Acknowledge efforts of extension agents	Yes	80.0	73.3	80.0	86.7	100.0
	No	20.0	26.7	20.0	13.3	0.0

### PHL and pest infestations

**Figure 7:**
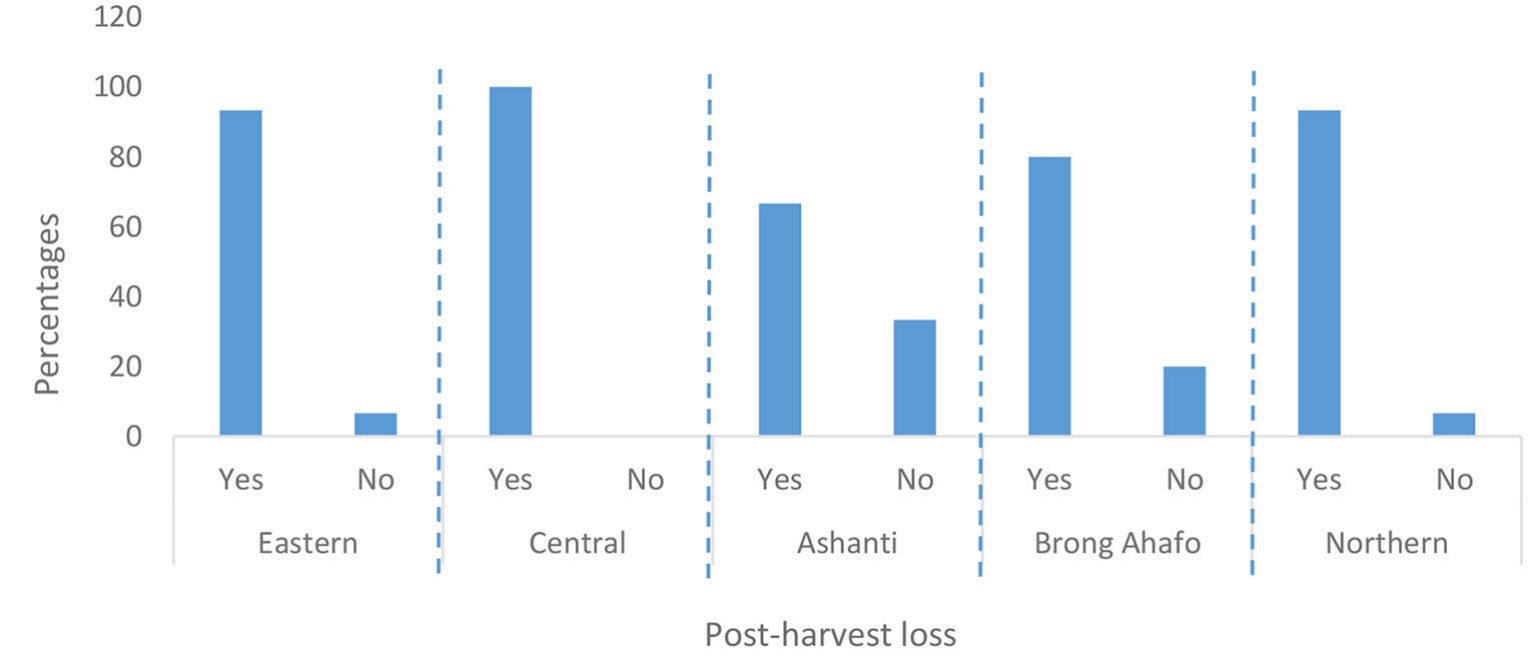
Percentage of maize farmers that observed post-harvest losses (PHL) in maize grain.

**Figure 8:**
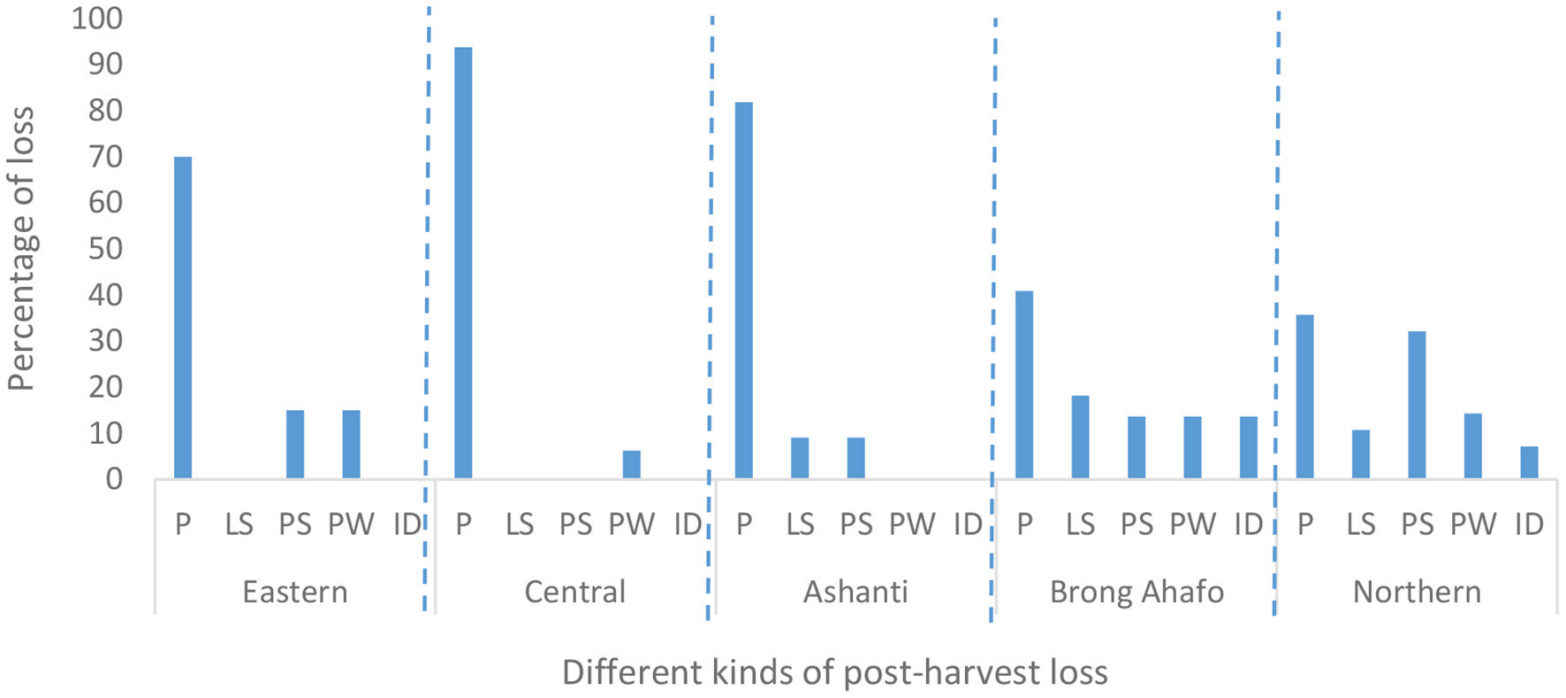
Different kinds of post-harvest loss (PHL) observed by maize farmers. P, pest infestation; LS, lack of storage facility; PS, poor storage facility; PW, poor weather; ID, improper drying.

**Figure 9:**
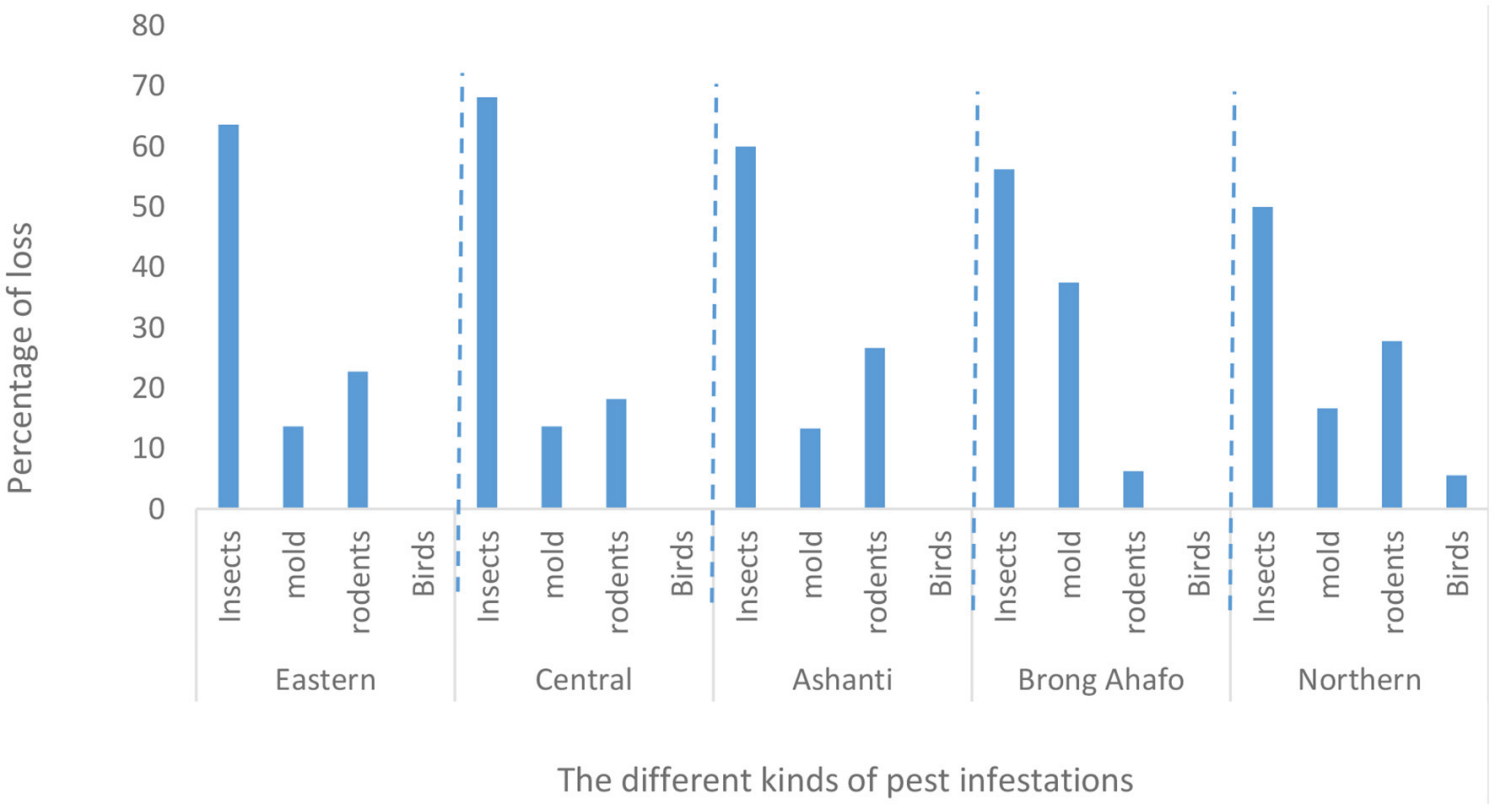
Loss (%) in maize grain due to different kinds of pest infestations in Ghana.

More than 67% of farmers in all five regions experienced PHL (Figure 7). This means farmers in all the five regions experience some form of PHL. PHL was predominantly caused by pest infestation (36–94% of farmers, Figure 8). Pest infestation was due mostly to insects, followed by rodents and then molds (Figure 9). *Sitophilus zeamais* was the only insect that infested the grain (Table 2). The observance of *S. zeamais* in stored grain is worrying because of its economic importance (grain loss). Such grain loss experienced by farmers may result in food insecurity and loss of income. The *S. zeamais* infestation may have been caused by delayed harvest practiced by farmers and poor storage facilities. Early harvest of grain reduces weevil infestation which begins from the field. PHL have similarly been reported in many studies (16, 18, 28–31). Molds infestation was not reported as severe compared to rodents. Also, the use of poor storage facilities worsened PHL.

## Conclusion

The number of male maize farmers surpassed their female counterparts. Males cultivated larger acreage of land compared with female farmers. The participated farmers were basically smallholder farmers since they cultivate <25 acres of land. Almost all the farmers used conventional practices in their activities except those who have access to locally manufactured machines. PHL was generally caused by maize weevils or rodents but not molds. Farmers used synthetic insecticides and plant material to control maize weevils. Generally, the knowledge of farmers on molds and mycotoxin effects was good, but was exceptional in NR. The efforts of the AEAs were greatly acknowledged in all the regions.

## Data availability statement

The raw data supporting the conclusions of this article will be made available by the authors, without undue reservation.

## Ethics statement

Ethical approval for this study and written informed consent from the participants of the study were not required in accordance with local legislation and national guidelines.

## Author contributions

KR and BD designed the study. BD implemented the survey, collected and analyzed data, and drafted the manuscript. KR supervised the study and revised the manuscript. All authors contributed to the article and approved the submitted version.

## Funding

This work was funded by the Feed the Future Ghana Agriculture Technology Transfer Project under the International Fertilizer Development Center (USAI-Ghana) in partnership with ISU.

## Conflict of interest

The authors declare that the research was conducted in the absence of any commercial or financial relationships that could be construed as a potential conflict of interest.

## Publisher's note

All claims expressed in this article are solely those of the authors and do not necessarily represent those of their affiliated organizations, or those of the publisher, the editors and the reviewers. Any product that may be evaluated in this article, or claim that may be made by its manufacturer, is not guaranteed or endorsed by the publisher.
