# Supplementary material for: Pre-harvest and post-harvest farmer experiences and practices in five maize growing regions in Ghana
Source: Front Nutr. 2022 Aug 19;9:725815. doi: 10.3389/fnut.2022.725815 (PMC9437942; doi:10.3389/fnut.2022.725815)
Supplement: Supplementary file 1 [file Data_Sheet_1.pdf]

Pre-harvest and post-harvest farmer experiences and practices in five maize growing regions in Ghana

Appendix: Survey Instrument Used

A: General information

1. Date…………………………………………………
   Region…………………………………………
2. Name of village…………………………………………………………………
3. Name of District…………………………………………………………….
4. Name of interviewee…………………………………………………………
5. Gender (a) Male [ ] (b) Female [ ]
6. Age (a) under 18 [ ] (b) 18-25 [ ] (c) 25-40 (d) over 40
7. Education level …………………………………………………………………
   (0 = none, 1 = Primary, 2 = Secondary, 3 = College, 4 = none, 5 = other…………)
8. What type of activity are you involved in..
   (1 = farming, 2 = trader, 3 = consumer, 4 = both 1, 2, & 3, 5 = other……….)

B: Pre-Harvest losses

9. Do you observe any losses before harvest....................................(1 = Yes, 2 = No)
10. What are the losses…………………………………………………………
    (1 = birds damage, 2 = weather, 3 = insects, 4 = Rodents, 5 = other……………)
11. Per your estimation what is the quantity of the losses
    (1= less than 1%, 2 = 2%, 3 = above 5%).

C: Information on Farmers

12. Total area cultivated (Ha) (a) below 5 [ ] (b) 5-10 [ ] (c) 10-50 [ ] (d) above 50 [ ]
13. How is the harvesting done………… (1 = Hand, 2 = Combine, 3 = Other…….)
14. Days used in the harvesting……………………………………………………
    (1 = less than 4 days, 2 = one week, 3 = two weeks, 4 = more than 2 weeks)
15. Bags or kg of maize harvested last season ……………...
16. How is the shelling/threshing done……………………………………………..
    (1 = hand, 2 = mechanized, 3 = improvised device, 4 = Beating, 5 = others………)
17. Any losses per harvested (if any)...(1 = Yes, 2 = No)
18. What are the main reasons for post-harvest loses..
    (1 = Pest infestation, 2 = lack of storage, 3 = Poor storage, 4 = Poor weather, 5 = improper drying, 6 = other ....)

If the answer in (18) is 1 go to Question 19

19. What types of pest infestation..?
    (1 = insects, 2 = mold, 3 = rodent/mice, 4 = birds, 5 = other ……......)

If the answer in (19) is 1 go to Question 20

20. What types of insect..?
    (1 = maize weevils, 2 = larger grain borer, 3 = other …………………)
21. An average number of months maize is stored ………….………....................
    (1 = less than 1 month, 2 = three months, 3 = six months, 4 = one year, 5 = other…..)
22. How is the maize grain dried..?
    (1= No drying, 2= sun drying, 3 = solar drying, 4 = mechanical drying, 5 = other....)
23. After harvesting do you sort out foreign materials (e.g.: other seeds, ears, stones………………………… (1 = Yes, 2 = No)
24. After harvesting do you sort out infected/diseased seeds………...(1 = Yes, 2 = No)
25. After harvesting do you sort out broken seeds……………………(1 = Yes, 2 = No)
26. What method do you use for the sorting………………………………………
    (1 = hand picking, 2 = winnowing, 3 = mechanical, 4 = other…………..……)
27. What do you do with the damaged maize ears………………………………?
    (1= throw away, 2= domestic consumption, 3= animal feeds, 4= sell, 5 = other…)
28. At what level do you discard maize grain...?
    (Picture: 1 = when show sign of mold growth, 2 = when showing a clear sign of mold growth, 3 = when is total moldy, 4 = not discard, 5 = other ………)
29. What methods of discard ……………………………………………………?
    (1 = used as animal feeds, 2 = burning, 3 = burial, 4 = left in the field, 5 = other…)
30. How do you store the grains after harvest………………………………
    (1 = traditional granary, 2 = silos, 3 = bags, 4 = rented facility, 5 = other ……)
31. Are the grains treated before storage………………………………………
    (1 = insecticides, 2 = fungicides, 3 = pesticides, 4 = fumigants, 5 = other)
32. Do you have any knowledge about the effect of moldy maize…? (1 = Yes, 2 = No)
33. Have you previously heard of the word mycotoxins (aflatoxins)? (1 = Yes, 2 = No)
34. Are you aware of mycotoxins contamination in maize?………(1 = Yes, 2 = No)
35. Are you aware of the effects of mycotoxins on human and animals?.......................(1 = Yes, 2 = No)
36. In your view, where in the post-harvest maize value chain do the major losses occur?
    (1 = transport from field to home, 2 = drying, 3 = shelling, 4 = storage, 5 = transport to market, 6 = during marketing, 7 = other ……………………………)
37. Do you produce for consumption or for sale……………...... (1 = Yes, 2 = No, 3 = Both, 4 = Others……………..)
38. Are you visited by extension agents……………………(1 = Yes, 2 = No)
39. How frequently do they visit your farm……..... (1 = once per year, 2 = twice per year, 3 = thrice per year, 4 = other ……………….....)
40. Are the services of the extension agents useful/helpful to you…..(1 = Yes, 2 = No)
41. If 40 is **NO,** then what do you expect from these agents………………………
42. What services do the agents provide to you…………………………………….
43. Are you willing to offer your farm for my research studies next year...……. (1 = Yes, 2 = No).
